# Synthesis, Characterization and *In Vitro* Antibacterial Studies of Organotin(IV) Complexes with 2-Hydroxyacetophenone-2-methylphenylthiosemicarbazone (H_2_dampt)

**DOI:** 10.1155/2012/698491

**Published:** 2012-03-07

**Authors:** M. A. Salam, M. A. Affan, Ramkrishna Saha, Fasihuddin B. Ahmad, Norrihan Sam

**Affiliations:** ^1^Faculty of Resource Science and Technology, Universiti Malaysia Sarawak, 94300 Kota Samarahan, Sarawak, Malaysia; ^2^Department of Chemistry, Shahjalal University of Science and Technology, Sylhet 3114, Bangladesh

## Abstract

Five new organotin(IV) complexes of 2-hydroxyacetophenone-2-methylphenylthiosemicarbazone [H_2_dampt, (**1**)] with formula [RSnCl_*n-1*_(dampt)] (where R = Me, *n* = 2 (**2**); R = Bu, *n* = 2 (**3**); R = Ph, *n* = 2 (**4**); R = Me_2_, *n* = 1 (**5**); R = Ph_2_, *n* = 1 (**6**)) have been synthesized by direct reaction of H_2_dampt (**1**) with organotin(IV) chloride(s) in absolute methanol. The ligand (**1**) and its organotin(IV) complexes (**2–6**) were characterized by CHN analyses, molar conductivity, UV-Vis, FT-IR, ^1^H, ^13^C, and ^119^Sn NMR spectral studies. H_2_dampt (**1**) is newly synthesized and has been structurally characterized by X-ray crystallography. Spectroscopic data suggested that H_2_dampt (**1**) is coordinated to the tin(IV) atom through the thiolate-S, azomethine-N, and phenoxide-O atoms; the coordination number of tin is five. The *in vitro* antibacterial activity has been evaluated against *Staphylococcus aureus, Enterobacter aerogenes, Escherichia coli*, and *Salmonella typhi*. The screening results have shown that the organotin(IV) complexes (**2–6**) have better antibacterial activities and have potential as drugs. Furthermore, it has been shown that diphenyltin(IV) derivative (**6**) exhibits significantly better activity than the other organotin(IV) derivatives (**2–5**).

## 1. Introduction

Thiosemicarbazones and their metal complexes have received considerable attention in chemistry and biology, primarily because of their marked and various biological properties [1–3]. The pharmacological profiles of 2-formyl, 2-acetyl, and 2-benzoylpyridine thiosemicarbazones have been investigated [4]. Seena and Kurup [5] have synthesized and characterized dioxomolybdenum(IV) complexes with 2-hydroxyacetophenone-*N*(4)-cyclohexyl and *N*(4)-phenyl thiosemicarbazone which suggested that the Mo(IV) complex is pentacoordinated [5]. For the past few years, studies of the coordination chemistry of thiosemicarbazone involved complexes with transition metal ions [6–8]. Organotin(IV) complexes have been the subject of interest for some time because of their biomedical and commercial applications including *in vitro* and *in vivo* antitumor activity [9, 10]. Many organotin(IV) complexes have been found to be as effective as or even better than traditional anticancer drugs [11–14]. Organotin(IV) chelates with nitrogen, sulfur, and oxygen donor ligands have gained attention during the last few years [15]. The coordination chemistry of tin is extensive with various geometries and coordination numbers known for both inorganic and organometallic complexes [16, 17]. In our previous work, we have reported some new organotin(IV) complexes with heterocyclic-*N*(4)-cyclohexylthiosemicarbazone ligands [18, 19]. The results revealed that thiosemicarbazones derived from 2-benzoylpyridine and 2-acetylpyrazine and their tin(IV)/organotin(IV) complexes have been characterized by different spectroscopic techniques. From the literature survey, the studies on the organotin(IV) complexes derived from substituted thiosemicarbazone ligands containing ONS-donor atoms are still lacking. To the best of our knowledge, there was no report on the organotin(IV) complexes of the 2-hydroxyacetophenone-2-methylphenylthiosemicarbazone. In this view, we have synthesized a series of organotin(IV) complexes with 2-hydroxyacetophenone-2-methylphenylthiosemicarbazone. These complexes have been characterized by elemental analysis, ^1^H, ^13^C, and ^119^Sn NMR spectroscopy. X-ray crystal structure of 2-hydroxyacetophenone-2-methylphenylthiosemicarbazone (**1**) is also described. Their biological activity data has also been reported.

## 2. Experimental

### 2.1. Materials and Methods

All reagents were purchased from Fluka, Aldrich, and JT Baker. All solvents were purified according to standard procedures [20]. UV-Vis spectra were recorded in CHCl_3_ solution with a Perkin Elmer Lambda 25 UV-Visible spectrometer. Infrared spectra were recorded on KBr discs using a Perkin Elmer Spectrum GX Fourier-Transform spectrometer in the range 4000–370 cm^−1^ at room temperature. ^1^H, ^13^C, and ^119^Sn NMR spectra were recorded on a JEOL 500 MHz-NMR spectrometer; chemical shifts were given in ppm relative to SiMe_4_ and SnMe_4_ in CDCl_3_ solvent. CHN analyses were obtained with a Flash EA 1112 series CHN elemental analyzer. Molar conductivity measurements were carried out with Jenway 4510 conductivity meter using DMF solvent mode.

### 2.2. Synthesis of 2-Hydroxyacetophenone-2-Methylphenylthiosemicarbazone (H_2_dampt) **(1)**

**Scheme 1:**
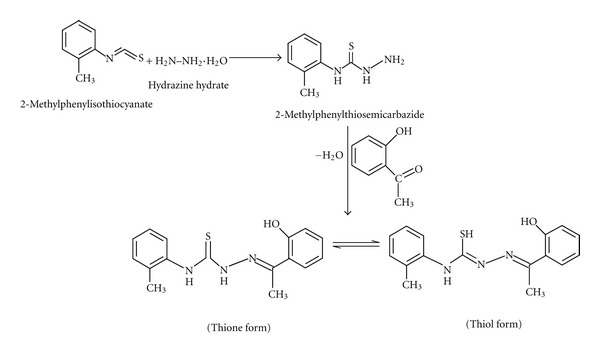
Synthesis of 2-hydroxyacetophenone-2-methylphenylthiosemicarbazone (H_2_dampt) ligand (**1**).

The 2-methylphenylisothiocyanate (0.746 g, 5 mmol) and hydrazine hydrate (0.253 g, 5 mmol), each dissolved in 10 mL ethanol, were mixed with constant stirring. The stirring was continued for 30 min and the white product, 2-methylphenylthiosemicarbazide, formed was washed with ethanol and dried *in vacuo*. A solution of the isolated 2-methylphenylthiosemicarbazide (0.540 g, 3 mmol) in 10 mL methanol was then refluxed with a methanolic solution of 2-hydroxyacetophenone (0.408 g, 3 mmol) for 5 h after adding 1-2 drops of glacial acetic acid (Scheme 1). On cooling the solution to room temperature, light-yellow microcrystals were separated and washed with methanol. The microcrystals were recrystallized from methanol and dried *in vacuo* over silica gel. Yield: 0.74 g, 78%: M.p.: 178–180°C: UV-Visible (CHCl_3_) *λ*_max⁡/nm_: 226, 318, 359: FT-IR (KBr disc, cm^−1^) *ν*_max⁡_: 3175 (s, OH), 3000 (s, NH), 1583 (m, C=N), 1298 (m, C–O), 943 (m, N–N), 1371, 861 (w, C=S). ^1^H NMR (CDCl_3_) *δ*: 10.82 (s, 1H, OH), 9.02 (s, 1H, N–H), 7.31–7.25 (m, 8H, phenyl ring), 2.56 (s, 3H, N=C–CH_3_), 2.29 (s, 3H, CH_3_), 1.19 (s, 1H, SH). ^13^C NMR (CDCl_3_) *δ*: 185.20 (NH–C=S), 165.32 (C=N), 145.30–136.21 (aromatic ring), 10.45 (CH_3_). Anal. Calc. for C_16 _H_17_N_3_OS: C, 64.21; H, 5.73; N, 14.04%. Found: C, 64.17; H, 5.67; N, 14.01%.

### 2.3. Synthesis of [MeSnCl(dampt)] **(2)**

**Scheme 2:**
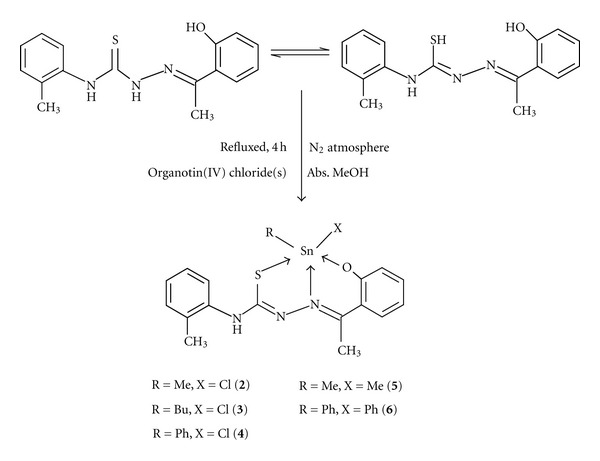
Reaction scheme for the synthesis of organotin(IV) complexes (**2–6**).

H_2_dampt (0.299 g, 1.0 mmol) was dissolved in absolute methanol (10 mL) in a Schlenk round bottom flask under a nitrogen atmosphere. Then, a methanolic solution of methyltin(IV) trichloride (0.24 g, 1.0 mmol) was added dropwise. The resulting reaction mixture was refluxed for 4 h (Scheme 2) and cooled to room temperature. The microcrystals were filtered off, washed with a small amount of cold methanol, and dried *in vacuo* over silica gel. Yield: 0.41 g, 76 %: Mp.: 222–224°C: Molar conductance (DMF) Ω^-1 ^cm^2^ mol^−1^: 7.1: UV-Visible (CHCl_3_) *λ*_max⁡/nm_: 262, 328, 367, 384: FT-IR (KBr, cm^−1^) *ν*_max⁡_: 3378 (s, NH), 1595 (m, C=N–N=C), 1268 (m, C–O), 1026 (w, N–N), 1306, 822 (m, C–S), 612 (w, Sn–C), 570 (w, Sn–O), 449 (w, Sn–N). ^1^H NMR (CDCl_3,_^2^J[^119^Sn, ^1^H]) *δ*: 9.08 (s, 1H, N–H), 7.26–6.94 (m, 8H, phenyl ring), 2.95 (s, 3H, N=C–CH_3_), 2.30 (s, 3H, CH_3_), 1.09 (s, 3H, Sn–CH_3_), [74.4 Hz]. ^13^C NMR (CDCl_3_) *δ*: 180.55 (N=C–S), 170.88 (C=N), 144.35–135.60 (aromatic ring), 18.70 (CH_3_), 12.80 (Sn–CH_3_). ^119^Sn NMR (CDCl_3_) *δ*: −168.5. Anal. Calc. for C_17_H_18_N_3_SOSnCl: C, 43.76; H, 3.88; N, 9.00%. Found: C, 43.71; H, 3.82; N, 8.95%.

The other complexes (**3–6**) were synthesized using a similar procedure to organotin(IV) complex (**2**) using appropriate organotin(IV) chloride(s) (Scheme 2).

### 2.4. Synthesis of [BuSnCl(dampt)] **(3)**

Yield: 0.43 g, 74%: Mp.: 226–228°C: Molar conductance (DMF) Ω^-1 ^cm^2^ mol^−1^: 9.1: UV-Visible (CHCl_3_) *λ*_max⁡/nm_: 262, 328, 382, 397: FT-IR (KBr, cm^−1^) *ν*_max⁡_: 3374 (s, NH), 1599 (m, C=N–N=C), 1254 (m, C–O), 1014 (w, N–N), 1299, 835 (m, C–S), 605 (w, Sn–C), 568 (w, Sn–O), 443 (w, Sn–N). ^1^H NMR (CDCl_3_) *δ*: 9.07 (s, 1H, N–H), 7.25–7.97 (m, 8H, phenyl ring), 2.62 (s, 3H, N=C–CH_3_), 2.30 (s, 3H, CH_3_), 2.28–2.15 (t, 2H, Sn–CH_2_–CH_2_–CH_2_–CH_3_), 2.14–1.73 (m, 2H, Sn–CH_2_–CH_2_–CH_2_–CH_3_), 1.24–1.22 (m, 2H, Sn–CH_2_–CH_2_–CH_2_–CH_3_), 0.99–0.86 (t, 3H, Sn–CH_2_–CH_2_–CH_2_–CH_3_). ^13^C NMR (CDCl_3_) *δ*: 178.99 (N=C–S), 168.36 (C=N), 145.20–136.22 (aromatic ring), 32.78, 26.31, 24.18, 20.11 (Sn–CH_2_–CH_2_–CH_2_–CH_3_), 16.44 (CH_3_). ^119^Sn NMR (CDCl_3_) *δ*: −149.6. Anal. Calc. for C_20_H_24_N_3_SOSnCl: C, 45.10; H, 4.54; N, 7.88%. Found: C, 45.00; H, 4.51; N, 7.81%.

### 2.5. Synthesis of [PhSnCl(dampt)] **(4)**

Yield: 0.48 g, 79%: Mp.: 218–220°C: Molar conductance (DMF) Ω^-1 ^cm^2^ mol^−1^: 3.11: UV-Visible (CHCl_3_) *λ*_max⁡/nm_: 263, 335, 381, 410: FT-IR (KBr, cm^−1^) *ν*_max⁡_: 3184 (s, NH), 1598 (m, C=N–N=C), 1240 (m, C–O), 1035 (w, N–N), 1300, 838 (m, C–S), 601 (w, Sn–C), 522 (w, Sn–O), 471 (w, Sn–N). ^1^H NMR (CDCl_3_) *δ*: 9.01 (s, 1H, N–H), 7.24-6.95 (m, 13H, phenyl ring), 2.76 (s, 3H, N=C–CH_3_), 2.29 (s, 3H, CH_3_). ^13^C NMR (CDCl_3_) *δ*: 180.12 (N=C–S), 173.97 (C=N), 144.84–136.60 (aromatic ring), 16.88 (CH_3_). ^119^Sn NMR (CDCl_3_) *δ*: −174.73. Anal. Calc. for C_22_H_20_N_3_SOSnCl: C, 49.98; H, 3.81; N, 7.94%. Found: C, 48.92; H, 3.77; N, 7.90%.

### 2.6. Synthesis of [Me_2_Sn(dampt)] **(5)**

Yield: 0.41 g, 78%: Mp.: 210–212°C: Molar conductance (DMF) Ω^−1 ^cm^2^ mol^−1^: 5.2: UV-Visible (CHCl_3_) *λ*_max⁡/nm_: 266, 338, 378, 414: FT-IR (KBr, cm^−1^) *ν*_max⁡_: 3320 (s, NH), 1605 (m, C=N–N=C), 1252 (m, C–O), 1036 (w, N–N), 1300, 832 (m, C–S), 603 (w, Sn–C), 523 (w, Sn–O), 499 (w, Sn–N). ^1^H NMR (CDCl_3,_^2^J[^119^Sn, ^1^H]) *δ*: 9.08 (*s*, 1H, N–H), 7.26–6.94 (m, 8H, phenyl ring), 2.98 (s, 3H, N=C–CH_3_), 2.30 (s, 3H, CH_3_), 0.98 (s, 3H, Sn–CH_3_), [77.5 Hz]. ^13^C NMR (CDCl_3, _[^1^J (^13^C–^119^Sn]) *δ*: 181.10 (N=C–S), 178.45 (C=N), 145.68–137.20 (aromatic ring), 17.5 (CH_3_), 14.97 (Sn–CH_3_) [557 Hz]. ^119^Sn NMR (CDCl_3_) *δ*: −182.45. Anal. Calc. for C_18_H_21_N_3_SOSn: C, 48.54; H, 4.74; N, 9.41%. Found: C, 48.50; H, 4.71 N, 9.38%.

### 2.7. Synthesis of [Ph_2_Sn(dampt)] **(6)**

Yield: 0.48 g, 75%: Mp.: 258–260°C: Molar conductance (DMF) Ω^−1 ^cm^2^ mol^−1^: 8.17: UV-Visible (CHCl_3_) *λ*_max⁡/nm_: 268, 327, 373, 402: FT-IR (KBr, cm^−1^) *ν*_max⁡_: 3383 (s, NH), 1592 (m, C=N–N=C), 1265 (m, C–O), 1039 (w, N–N), 1307, 821 (m, C–S), 601 (w, Sn–C), 570 (w, Sn–O), 448 (w, Sn–N). ^1^H NMR (CDCl_3_) *δ*: 9.02 (*s*, 1H, N–H), 7.30–6.97 (m, 13H, phenyl ring), 2.67 (s, 3H, N=C–CH_3_), 2.29 (s, 3H, CH_3_). ^13^C NMR (CDCl_3, _[^1^J (^13^C–^119^Sn]) *δ*: 179.98 (N=C–S), 171.75 (C=N), 142.20–138.21 (aromatic ring), 15.88 (CH_3_) [546 Hz].^ 119^Sn NMR (CDCl_3_) *δ*: −185.32. Anal. Calc. for C_28_H_25_N_3_SOSn: C, 58.97; H, 4.41; N, 7.36%. Found: C, 58.92; H, 4.38; N, 7.30%.

### 2.8. Antibacterial Test

The synthesized ligand (**1**) and its organotin(IV) complexes (**2–6**) were screened *in vitro* for their antibacterial activity against *Staphylococcus aureus*, *Enterobacter aerogenes, Escherichia coli,* and *Salmonella typhi* bacterial strains using agar-well diffusion method [21]. Wells (size of well 6 mm in diameter) were dug in the media with the help of a sterile metallic borer with centers at least 24 mm. Eight-hour old bacterial inoculums containing 10^4^–10^6^ colony-forming units (CFU)/mL were spread on the surface of the nutrient agar using a sterile cotton swab. Recommended concentration of the test sample (200 mg/mL in DMSO) was introduced in the respective wells. Other wells supplemented with DMSO and reference drug (doxycycline) served as negative and positive controls, respectively. The plates were incubated immediately at 37°C for 20 h. Activity was determined by measuring the diameter of zones showing complete inhibition (mm). Growth inhibition was calculated with reference to the positive control.

## 3. Results and Discussion

### 3.1. Synthesis

2-Hydroxyacetophenone-2-methylphenylthiosemicarbazone (H_2_dampt) was synthesized by the condensation reaction of 2-hydroxyacetophenone and 2-methylphenylthiosemicarbazide in absolute methanol in 1 : 1 mole ratio. It has two tautomers within the structure, existing as either thione or thiol tautomer (Scheme 1). The present organotin(IV) complexes (**2–6**) were obtained by direct reaction of organotin(IV) chloride(s) and H_2_dampt (**1**) in absolute methanol under N_2_ atmosphere (Scheme 2). The physical properties and analytical data of H_2_dampt (**1**) and its organotin(IV) complexes (**2–6**) are given in the experimental section. All complexes (**2–6**) were stable under N_2_ atmosphere and soluble in CHCl_3_, CH_2_Cl_2_, DMF, DMSO, and MeCN solvents except methanol, ethanol, hexane, pentane, THF, and ether. The molar conductances values of the complexes (**2–6**) are 9.1–3.1 Ω^−1 ^cm^2^ mol^−1^, respectively, indicate that the complexes behave as nonelectrolytes [22].

### 3.2. UV-Visible Spectra

The UV-Vis spectra of ligand (**1**) and its organotin(IV) complexes (**2–6**) were carried out in CHCl_3_ (1 × 10^−4^ mol L^−1^) at room temperature. The free ligand (**1**) exhibited three absorption bands at 262, 318, and 359 nm assigned to the HOMO/LUMO transition of phenolic group, azomethine, and thiolate function, respectively [23]. After complexation, the UV-Vis spectra of the complexes (**2–6**) exhibited four absorption bands in the region at 262–268, 327–338, 367–382, and 384–414 nm, respectively. In the electronic spectra of the complexes (**2–6**), the intraligand transition is shifted to higher wavelength as a result of coordination. In the spectra of organotin(IV) complexes (**2–6**), one new absorption band appeared at 384–414 nm which is assigned to the ligand→metal charge transfer (LMCT) [24]. The shift of the *λ*_max⁡_ band from the ligand to the complex is supported by the coordination of ligand (**1**) to the tin(IV) ion.

### 3.3. IR Spectra

The IR spectrum of free ligand (**1**) showed absorption bands at 3175 and 3000 cm^−1^, which are due to the stretching vibrations of the OH and NH groups, respectively. The absorption bands at 1583, 1298, 943, and 1371, 861 cm^−1^are due to *ν*(C=N), *ν*(C–O), *ν*(N–N), and *ν*(C=S), respectively. Several significant changes with respect to the free ligand (**1**) bands on complexation suggest coordination through phenolic group, azomethine, and sulfur of the thiolic form of the ligand. The strong stretching band at 3375 cm^−1^ that corresponds to the *ν*(OH) group in the spectrum of ligand (**1**) has disappeared in the spectra of complexes (**2–6**) due to the deprotonation, indicating coordination through the phenolic oxygen to tin(IV) atom. The free ligand (**1**) showed a band at 1298 cm^−1^ which is due to *ν*(C–O). This band is shifted to lower wave numbers at 1240–1268 cm^−1^ in the complexes (**2–6**), indicating the coordination of O^−^ to the tin(IV) atom [25]. The newly formed *ν*(C=N–N=C) bond showed medium-to-strong absorption peaks in the range at 1592–1605 cm^–1^ in the spectra of the complexes (**2–6**), indicating coordination of azomethine nitrogen to tin(IV) atom [26]. A sharp band at 943 cm^−1^ is due to *ν*(N–N) for ligand (**1**) is shifted to higher frequencies at 1014–1039 cm^−1^ in the spectra of organotin(IV) complexes ((**2–6).** The increase in the frequency of this band in the spectra of complexes (**2–6**) due to an increase in the bond length again confirms coordination *via* the azomethine nitrogen atom [27]. The bands at 1371 and 861 cm^−1^ in the free ligand (**1**) due to *ν*(C=S) stretching vibrations are shifted to lower frequencies at 1299–1307 cm^–1^ and 821–838 cm^−1^ in the spectra of the complexes (2–6), suggesting coordination through the thiolate sulfur with tin(IV) atom [28]. The IR bands observed in the range at 570–522 cm^–1^ in the spectra of the complexes (**2–6**) suggest the presence of Sn–O bonding in their structure. The *ν*(Sn–C) and *ν*(Sn–N) bands are tentatively assigned to absorptions in the regions 612–601 cm^–1^ and 443–499 cm^–1^, respectively. Based on the infrared spectra analyses of ligand (**1**) and its organotin(IV) complexes (**2–6**), it was suggested that ligand (**1**) was coordinated to the tin(IV) core through the phenoxide-O, azomethine-N, and thiolato-S atoms.

### 3.4. ^1^H NMR Spectra

^1^H NMR spectrum of free ligand (**1**) showed resonance signals at 10.82, 9.02, 7.31–7.25, 2.56, 2.29, and 1.19 ppm are due to OH, NH, phenyl ring protons, N=C–CH_3_, CH_3_, and SH, respectively. After complexation, the resonance signal of OH proton was absent in the spectra of the complexes (**2–6**), indicating deprotonation of the phenolic proton and supported the phenolic oxygen atom was coordinated with tin(IV) atom. The resonance signal of SH is not found in the spectra of complexes (**2–6**) which suggested the deprotonation of the SH proton and confirming that the ligand coordinated to the tin(IV) in the thiolate form. The azomethine proton (N=C–CH_3_) signal appears at 2.56 ppm in the free ligand (**1**) which is shifted to high frequency at 2.98–2.62 ppm in the complexes (**2–6**), supporting the coordination of azomethine nitrogen to the central tin(IV) atom. The resonance signals for the protons of phenyl moiety of the ligand (**1**) were observed at 7.31–7.25 ppm, which is shifted to low frequency at 7.30–6.94 ppm in the complexes (**2–6**). This is due to the electron withdrawal tendency from the aromatic ring owing to coordination with tin(IV). The methyl group attached to the tin(IV) in complexes 2 and 5 gave a singlet at 1.09 and 0.98 ppm with ^2^J[^119^Sn,^ 1^H] coupling constant value equal to 74.4 and 77.5 Hz, respectively, supporting the five-coordinate environment around tin(IV) [29]. The three butyl groups attached to the tin(IV) moity in the organotin(IV) complex 3 gave four resonance signals, namely, 2.28–2.15 ppm (triplet, Sn–CH_2_–CH_2_–CH_2_–CH_3_), 2.14–1.73 ppm (multiplet, Sn–CH_2_–CH_2_–CH_2_–CH_3_), 1.24–1.22 ppm (multiplet, Sn–CH_2_–CH_2_–CH_2_–CH_3_), and 0.99–0.86 ppm (triplet, Sn–CH_2_–CH_2_–CH_2_–CH_3_). ^1^H NMR information also supported the IR data of the complexes (**2–6**).

### 3.5. ^13^C NMR Spectra

The ^13^C-{^1^H} NMR spectrum of free ligand (**1**) showed the resonance signals at 185.20, 165.32, 145.30–136.21, and 10.45 ppm are due to the *δ*(NH–C=S), *δ*(C=N), *δ* (aromatic ring carbon) and *δ*(CH_3_), respectively. After complexation, the carbon signals of the N=C–S group shifted to low frequency at 179.99–181.10 ppm in all the complexes (**2–6**) compared to ligand (**1**), indicating participation of the N=C–S group in coordination to tin(IV) atom. The chemical shifts of carbon in C=N and CH_3_ in the free ligand (**1**) were observed at 165.32 and 10.45 ppm which were shifted to high frequency at 168.36–178.45 and 16.44–18.70 ppm, respectively, in the complexes (**2–6**). This results supported the azomethine-N is coordinated to the tin(IV) atom [30]. After complexation, the *δ* value of carbon atoms in the aromatic ring did not have much change in the complexes (**2–6**) as compared to the free ligand. Besides, the butyl group attached to the organotin(IV) moiety in complex 3 gave four resonance signals at 32.78, 26.31, 24.18, and 20.11 ppm. In the ^13^C-{^1^H} NMR spectra of the organotin(IV) complexes **2** and **5**, a sharp singlet resonance signal appeared at 12.80 ppm [(Sn–CH_3_)] and 14.97 [Sn–(CH_3_)_2_] ppm, respectively [31]. In organotin(IV) compounds, the ^1^J[^119^Sn, ^13^C] value is an important parameter to assess the coordination number of the Sn atom. The calculated coupling constants for dimethyltin(IV) (4) and diphenyltin(IV) (5) compounds were found to be 557 and 546 Hz, which described the penta-coordinate environment about the Sn atom in these compounds [32]. All these statements are also supported by the ^1^H NMR spectra analyses.

### 3.6. ^119^Sn NMR Spectra

^119^Sn NMR spectra can be used as an indicator of the coordination number of the tin atom. ^ 119^Sn NMR of all the complexes (**2–6**) shows only one resonance signals in the range of −149.60 to −185.32 ppm. ^119^Sn NMR values are characteristic for the five-coordinated tin atom observed in the organotin(IV) complexes (**2–6**) [33–36].

### 3.7. X-Ray Crystallography Diffraction Analysis

**Figure 1:**
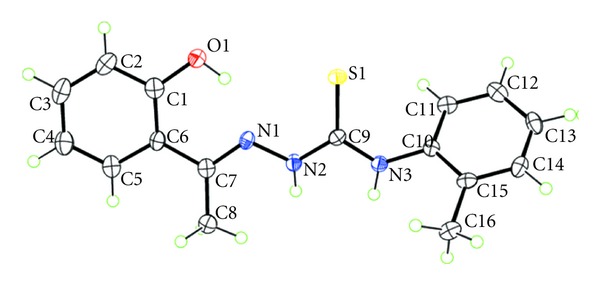
The molecular structure of H_2_dampt (**1**) showing the atom-labelling scheme and displacement ellipsoids at the 50% probability level.

**Figure 2:**
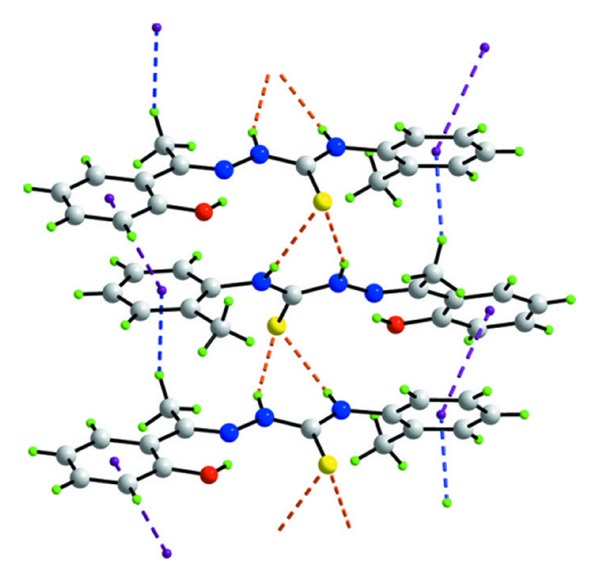
A view of the helical supramolecular chain aligned along the *b* axis in (I). The N–H···S hydrogen bonds are shown as orange dashed lines. Further stabilization to the chain is provided by C–H···*π* and *π*–*π* interactions, shown as blue and purple dashed lines, respectively.

The molecular structure of the ligand (**1**) with atom numbering scheme is depicted in Figure 1. The main crystal parameters are reported in Table 1. Selected bond lengths and bond angles are given in Table 2. The compound crystallizes into monoclinic crystal system with a space group of *P*2_1_/c. In the title substituted thiosemicarbazone, C_16_H_17_N_3_OS, the hydroxy- and methyl-substituted benzene rings form dihedral angles of 9.62 (12) and 55.69 (6)°, respectively, with the central CN3S chromophore (r.m.s. deviation = 0.0117 Å) in (C_16_H_17_N_3_OS) (Figure 1) and the OH– and Me-benzene rings are twisted as seen in the respective dihedral angles of 9.62 (12) and 55.69 (6)°. The almost coplanarity of the central atoms is ascribed to the formation of an intramolecular hydroxyl-O–H···N-imine hydrogen bond (Table 3). The N1–N2 bond length (1.375 Å) is closer to single bond length (1.45 Å) than to double bond length (1.25 Å) [37]. The C9–S1 bond distance (1.694 Å) is close to that expected of a C=S double bond (1.60 Å) [37] and the C7–N1 bond length (1.295 Å) is nearly the same as that of the C=N double bond (1.28 Å) [38]. These bond distances are in strong support of the existence of 2-hydroxyacetophenone-2-methylphenylthiosemicarbazo in the thione form in the solid state. The H atoms of the NH groups are *syn*, and the conformation about the N1=C7 double bond [1.295 (4) Å] is *E*. The *syn *arrangement in (C_16_H_17_N_3_OS)) contrast the *anti*arrangement often seen in such derivatives but is readily explained in terms of the intramolecular O–H···N-imine hydrogen bond in (C_16_H_17_N_3_OS)) by contrast to the normally observed intramolecular N–H···N-imine hydrogen bond [39, 40]. Helical supramolecular chains along the *b* axis dominate the crystal packing (Figure 2 and Table 3). These arise as a result of the thione-S interacting with both N–H atoms of a neighboring molecule thereby forming six-membered hydrogen-bond-mediated rings.

### 3.8. Antibacterial Activity

**Figure 3:**
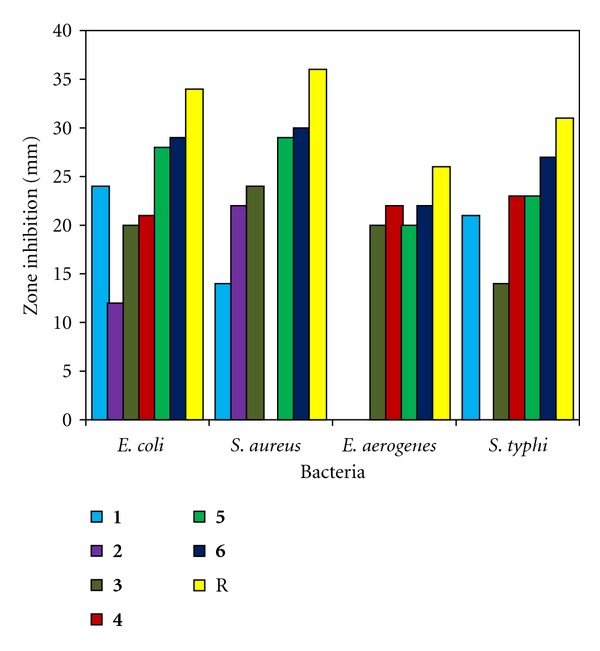
Antibacterial activity of compounds **1–6** against various bacteria.

The synthesized ligand (**1**) and its organotin(IV) complexes (**2–6**) were tested against *Escherichia coli*, *Staphylococcus aureus*, *Enterobacter aerogenes,* and *Salmonella typhi* bacterial strains for their antibacterial activity using agar-well diffusion method and data are shown in Table 4 and Figure 3. The doxycycline was used as a reference drug. The results showed that the substituted thiosemicarbazone ligand (**1**) possessed moderate antibacterial activity. The antibacterial studies of the compounds (**2–6**) showed relatively better activity against the selected bacteria than the free ligand (**1**), but low activities as compared to the reference drug. Among all the organotin(IV) derivatives, the bactericidal activities of **5** and **6** are fairly good. Complex **2** is the least active among all the organotin(IV) complexes, while complex **4** was found to be active against all the studied strains except *Staphylococcus aureus. *The most probable reason for this difference might be due to chelation which reduces the polarity of the central Sn atom because of the partial sharing of its positive charge with donor groups and possible *π*-electron delocalization within the whole chelating ring. As a result, the lipophilic nature of the central Sn atom increases, which favours the permeation of the complexes through the lipid layer of the cell membrane [41]. In addition, among the organotin(IV) complexes (**2–6**), complex (**6**) is found to be more active and it can be attributed to the presence of bulky phenyl groups which facilate binding to biological molecules *π*-*π* interactions.

## 4. Conclusion

The ligand (**1**) and its organotin(IV) complexes (**2–6**) have been synthesized and fully characterized by different spectroscopic techniques. The ligand (H_2_dampt) exists in thione form in a solid state but it takes on a thiol form when it is in solution. All organotin(IV) complexes (**2–6**) of H_2_dampt were proposed to be five coordinated and the ligand binds to the central tin(IV) atom in dinegative tridentate form. Single crystal X-ray analysis of newly synthesized ligand (**1**) has been reported. The *in vitro *antibacterial activities of the synthesized complexes against the selected bacterial strains have been established. All compounds have been found biologically active, while the studies have confirmed that compounds **5** and **6** are more active and have the potency to be used as antibacterial agents. Trials to obtain single crystals suitable for structure determination by X-ray crystallography were in vain due to the amorphous nature of the complexes.

## Figures and Tables

**Table 1:** Summary of crystal data and structure refinement parameters for ligand (1).

Compound	H_2_dampt (**1**)
Empirical formula	C_16_H_17_N_3_OS
Formula weight	299.39
Temperature (K)	100 (2)
Wavelength (Å)	0.71073
Crystal system	Monoclinic
Space group	*P*2_1_/c
Unit cell dimensions	
a (Å)	14.6966(8)
b (Å)	7.3586(4)
c (Å)	14.0926(8)
*α* (°)	90.00
*β* (°)	94.358(5)
*γ* (°)	90.00
Volume (Å^3^)	1519.66(15)
Z	4
Calculated density (mg/m3)	1.309
Radiation type *λ* (Å)	Mo K∖a
F (000)	632
Crystal size (mm)	0.30 × 0.1 × 0.05
Crystal colour	Light-yellow
Scan range *θ* (°)	2.8–29.3
Absorption coefficient (*μ*) (mm-1)	0.225
Max. and min. transm	1.00 and 0.419
Goodness of fit on F2	0.995
Data/restrains/ parameters	3375/3/201
Final R indices [I > 2*σ*(I)]	*R*_1_ = 0.0599, *wR*_2_ = 0.1324
R indices (all data)	*R*_1_ = 0.110, *wR*_2_ = 0.1738

**Table 2:** Selected bond lengths (Å) and bond angles (°) of ligand [H_2_dampt] (**1**).

Bond lengths (Å)			
S1–C9	1.694 (3)	O1–C1	1.357 (4)
N1–C7	1.295 (4)	N1–N2	1.375 (3)
N2–C9	1.352 (4)	N3–C9	1.344 (3)
C7–C8	1.500 (4)	C6–C7	1.473 (4)

Bond angles (°)			

C7–N1–N2	119.0 (2)	C9–N2–N1	120.6 (2)
C9–N3–C10	127.7 (2)	N3–C9–N2	113.2 (2)
N3–C9–S1	124.3 (2)	N2–C9–S1	122.4 (2)
O1–C1–C2	116.8 (3)	O1–C1–C6	123.2 (3)

**Table 3:** Hydrogen-bond geometry (Å, °)

*D-*H···*A *	*D-*H	H···*A *	*D*···*A *	*D-*H···*A *
O1–H1o···N1	0.84 (1)	1.81 (2)	2.551 (3)	145 (3)
N2–H2n···S1^i^	0.88 (1)	2.51 (2)	3.323 (2)	154 (3)
N3–H3n···S1^i^	0.88 (1)	2.49 (2)	3.286 (3)	151 (2)
C8–H8b···Cg1^i^	0.98	2.59	3.501 (3)	155
Symmetry codes: *–x* + 1, *y +* 1/2, −*z* + 1/2.				

**Table 4:** Antibacterial activity^a,b^ of the free ligand (**1**) and its organotin(IV) complexes 2–6 (inhibition zone in mm).

Bacterium	Clinical implication	Zone of Inhibition (mm)						
		**(1)**	**(2)**	**(3)**	**(4)**	**(5)**	**(6)**	**R**
*Escherichia coli *	Infection of wounds, urinary tract, and dysentery	24	12	20	21	28	29	34
*Staphylococcus aureus*	Food poisoning, scaled skin syndrome, endocarditis	14	22	24	—	29	30	36
*Enterobacter aerogenes*	Lower respiratory tract infections, skin and soft-tissue infections	—	—	20	22	20	22	26
*Salmonella typhi*	Typhoid fever, localized infection	21	—	14	23	23	27	31

^a^ *In vitro*, agar-well diffusion method, conc. 200 mg/mL of DMSO. ^b^Reference drug (R), doxycycline, dash indicate inactivity.
